# A case report of hereditary spherocytosis complicated by massive splenomegaly and cholelithiasis

**DOI:** 10.1093/jscr/rjaf602

**Published:** 2025-08-12

**Authors:** Qi Sun, Yang Yu, Lei Song, Lu Liang

**Affiliations:** Affiliated Baotou Clinical College of Inner Mongolia Medical University, No. 61 Hucheng Road, Donghe District, Baotou City, Inner Mongolia Autonomous Region 014040, China; Hepatobiliary and Splenic Surgery, Baotou Central Hospital, The Nei Monggol Autonomous Region, No. 61 Hucheng Road, Donghe District, Baotou City, Inner Mongolia Autonomous Region 014040, China; Hepatobiliary and Splenic Surgery, Baotou Central Hospital, The Nei Monggol Autonomous Region, No. 61 Hucheng Road, Donghe District, Baotou City, Inner Mongolia Autonomous Region 014040, China; Hepatobiliary and Splenic Surgery, Baotou Central Hospital, The Nei Monggol Autonomous Region, No. 61 Hucheng Road, Donghe District, Baotou City, Inner Mongolia Autonomous Region 014040, China

**Keywords:** hereditary spherocytosis, massive splenomegaly, cholelithiasis, combined surgery

## Abstract

Here, we report the case of a 53-year-old male patient with hereditary spherocytosis (HS) and a 10-year course of disease, presenting with fatigue, jaundice, abdominal discomfort, massive splenomegaly (spleen size: 35 × 20 × 10 cm, weight: 10 kg), and cholelithiasis. The patient had a positive family history, with his father and daughter exhibiting similar symptoms. Combined splenectomy and cholecystectomy significantly alleviated hemolytic jaundice, anemia, and gallstone-related symptoms. HS is an autosomal dominant disorder affecting erythrocyte membranes. Splenectomy effectively improves hemolytic anemia, while concurrent cholecystectomy is necessary to comprehensively manage cholelithiasis. This case highlights the clinical value of combined surgery for HS-related complications, emphasizing that personalized comprehensive treatment optimizes prognosis.

## Introduction

Hereditary spherocytosis (HS) is an autosomal dominant hereditary disorder caused by mutations in genes encoding erythrocyte membrane proteins, characterized by a reduction in the stability of red blood cell membranes, spheroidal transformation, and accelerated splenic destruction. This disease manifests clinically as hemolytic anemia, jaundice, splenomegaly, and cholelithiasis, with notable geographic variations in prevalence, reaching 1:2000 in Northern European populations while remaining relatively rare in Asian cohorts. Although splenectomy effectively alleviates hemolytic symptoms, chronic abnormalities in compensatory hemolysis-induced bilirubin metabolism frequently lead to gallstone formation, with 30%–60% of adult HS patients developing concomitant gallbladder stones. The coexistence of these conditions may exacerbate organ dysfunction and impair quality-of-life.

Current clinical guidelines recommend splenectomy as first-line therapy for patients with moderate-to-severe HS. However, the necessity for concurrent cholecystectomy in cases complicated by gallstones remains controversial. Some scholars argue that isolated splenectomy may accelerate postoperative gallstone-related complications, whereas combined surgery, despite resolving multi-system issues in a simultaneous manner, requires further clinical evidence to validate its safety and long-term efficacy in adult patients with HS. In addition, the management of massive splenomegaly (defined as a splenic weight > 1000 g or length > 20 cm) with biliary calculi necessitates multidisciplinary collaboration and perioperative optimization, yet relevant reports remain scarce in clinical practice.

Here, we report the case of an adult patient with a 10-year disease course of HS presenting with massive splenomegaly (10 kg) and multiple gallbladder stones, a combination demonstrating remarkable clinical significance. The implementation of combined splenectomy and cholecystectomy resulted in significant postoperative improvements in both hemolytic parameters and biliary symptoms, providing critical insights for therapeutic decision-making in such complex cases. In this case report, we explore coordinated management strategies for HS-related complications while accumulating clinical experience to evaluate surgical indications and optimize perioperative care for combined procedures.

## Case report

**Figure 1:**
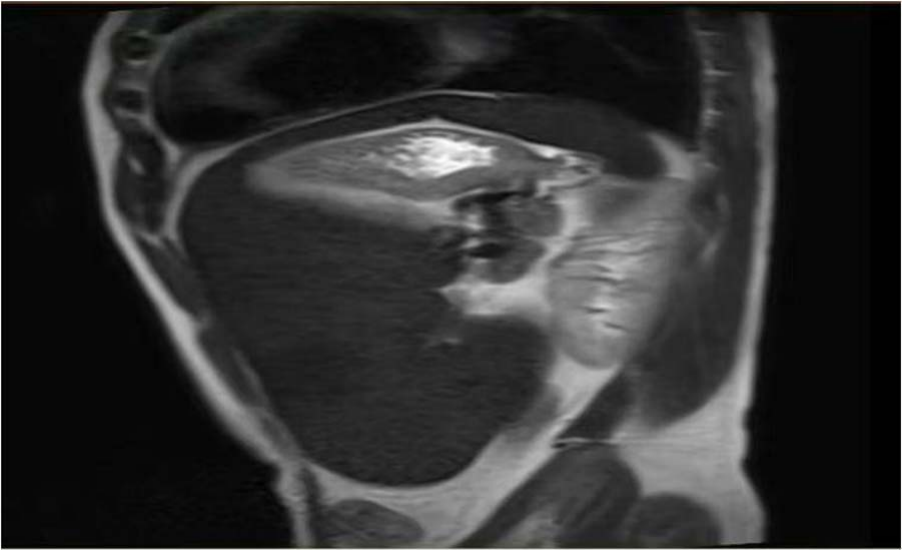
Imaging examination.

**Figure 2:**
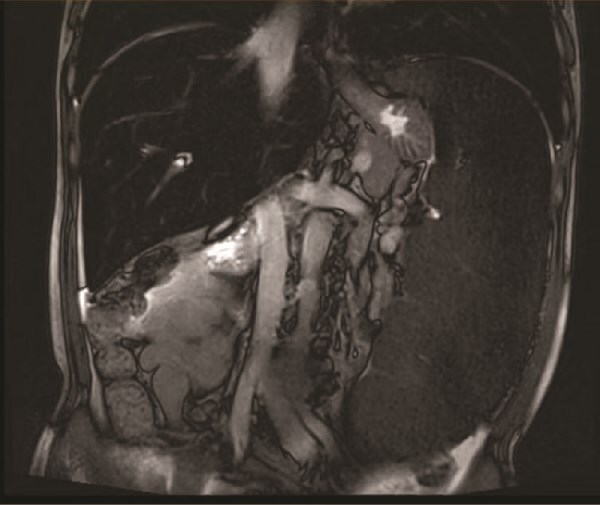
Imaging examination.

On the 19 August 2024, a 53-year-old man reported to our hospital complaining of “fatigue, jaundice, and abdominal discomfort for 10 years.” His hemolytic jaundice had been present for almost a decade. A family history of hereditary hemolytic jaundice was identified. His daughter and father both experienced similar ailments. Physical evaluation revealed that the skin and sclera were both stained by moderate yellowness. The left abdomen was full, and the spleen was hard and immovable, located 15 cm below the costal border. A computed tomography scan revealed that the spleen was 27.1 cm in length, 7.1 cm in thickness, and located 17.0 cm below the costal border (Figs. 1 and 2). The gallbladder measured 12.0 × 3.2 cm and contained several stones.

**Figure 3:**
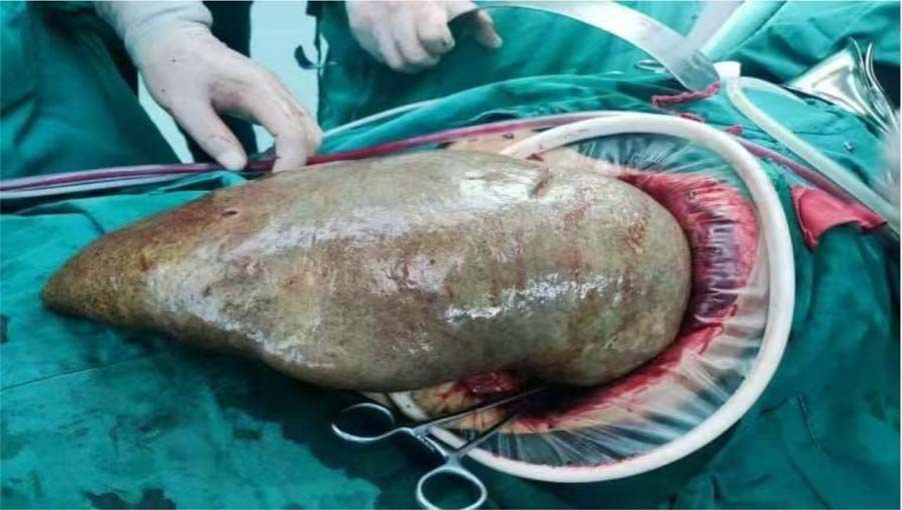
Surgical specimen.

**Figure 4:**
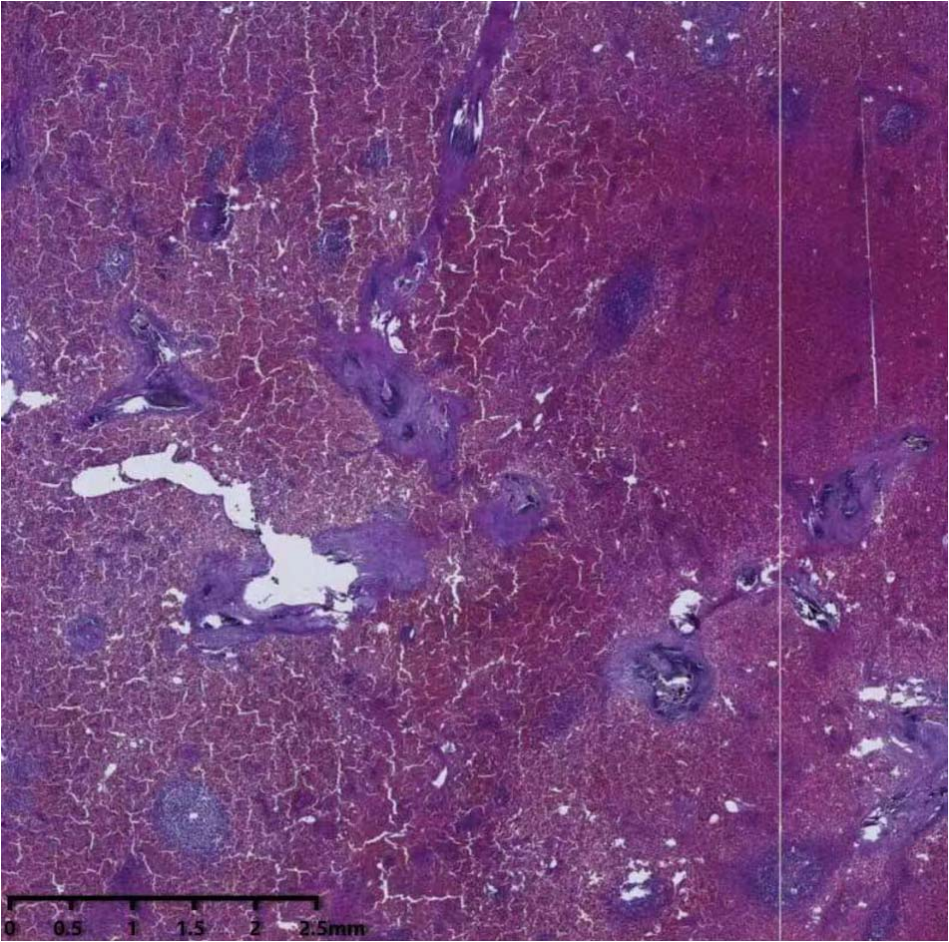
Histopathological section.

Because the patient exhibited evident signs of large splenomegaly, which was exerting a significant negative impact on his quality-of-life, we performed surgery on the 30 August 2024. Upon entering the abdominal cavity, we found no evidence of abnormalities in the liver, stomach, colon, small intestine, or mesentery. The spleen was enlarged, measuring ~35 × 20 × 10 cm^3^, with a pale appearance. The gallbladder measured ~10 × 6 × 4 cm^3^. Careful dissection of Calot’s triangle revealed the cystic duct diameter to be ~0.3 cm while that of the common bile duct was ~0.6 cm. The gallbladder was successfully removed. The splenocolic ligament was gradually dissected from the lower pole of the spleen toward the splenic hilum, exposing the splenic vessels and pancreatic tail. The gastrosplenic ligament was then dissected and exposed superiorly. Due to the massive size of the spleen, a decision was made to convert to open surgery. A midline upper abdominal incision, ~20 cm in length, was made, and the abdominal cavity was entered layer-by-layer. Then, we inserted a wound protector. Next, the short gastric vessels were individually dissected, clamped, and divided. The superior pole of the spleen was elevated, and the splenic ligaments were dissected, including the posterior splenorenal ligament. The dissection was completed by fully separating the splenic hilum, and the hilar tissues and vessels were divided sequentially to completely mobilize the spleen. The specimen was then removed intact. Subsequently, the abdominal cavity and operative field were thoroughly irrigated with warm saline. Inspection of the gallbladder bed, splenic fossa, gastric fundus, and vascular stumps confirmed no bleeding. Surgical gauze counts were correct. A 28# drainage tube was placed in the splenic fossa and exteriorized through the left trocar site. Another 28# drainage tube was placed in the Foramen of Winslow and exteriorized through the right trocar site. The abdomen was then closed layer-by-layer. Pathological examination (Fig. 3) revealed that the spleen was ~34 × 18.5 × 10 cm^3^ in size. Furthermore, the spleen had a smooth, slightly thickened capsule, a soft, grayish-red cut surface, vascular dilatation, congestion, and a few grayish-white streaks on the cut surface. The grayish-green gallbladder measured ~10 × 5 × 3 cm^3^, and there were several stones visible within. Microscopic analysis revealed thickening of the splenic capsule, widening of the splenic trabeculae, visible crystalline substances, a marked increase in red pulp and a reduction in white pulp, splenic cord congestion, splenic sinus dilatation, thickening of the vascular wall with fibrosis, and foreign body giant cell reaction (Fig. 4). The lymph nodes of the gallbladder showed signs of reactive hyperplasia. The patient was duly diagnosed with HS, hemolytic jaundice, hemolytic anemia, massive splenomegaly, and cholelithiasis with chronic cholecystitis.

## Discussion

HS is an autosomal dominant condition of red blood cell membranes [1] and is typified by an increase in the frequency of spherical red blood cells in the peripheral circulation. Due to their increased fragility and propensity to rupture, these aberrant red blood cells can cause several clinical symptoms, including cholelithiasis, splenomegaly, hemolytic anemia, jaundice, and hyperbilirubinemia [2]. The spleen is the primary organ responsible for the destruction of red blood cells, and while splenectomy cannot reverse the genetic mutation causing this disease, it can significantly alleviate symptoms, such as hemolytic anemia [3]. Eber *et al*. [4] classified HS into mild, moderate, and severe classifications based on hematological and other examination indicators. For mild cases or those without obvious symptoms, splenectomy may not be necessary; however, surgical intervention is required for moderate and severe cases. Furthermore, some researchers compared transfusion therapy with surgical treatment for patients with HS. Analysis showed that although transfusion therapy could significantly increase red blood cell counts and hemoglobin concentrations, repeated transfusions were required to maintain efficacy. In contrast, patients who underwent splenectomy recovered well postoperatively without requiring further transfusions, and symptoms such as anemia and jaundice were effectively alleviated. This not only improved quality-of-life but also reduced the wastage of blood resources. Therefore, total splenectomy remains the optimal treatment for HS at present. However, the cholelithiasis complication may exacerbate the clinical symptoms of certain patients. If a patient also has cholelithiasis, a splenectomy can significantly relieve hemolytic anemia and the symptoms associated with this condition; nevertheless, a single splenectomy cannot address every issue. In this instance, a combined splenectomy and cholecystectomy treatment strategy is worthwhile [5], since the latter can successfully alleviate the symptoms of cholelithiasis and avoid complications associated with the gallbladder.

In this case report, we describe the clinical management of a 53-year-old man with HS. He first complained of exhaustion, jaundice, and stomach pain. Following examination, the patient’s 10-year history of lethargy, jaundice, and stomach pain was determined to be caused by HS. Clinical management included cholecystectomy and splenectomy. The patient’s hemolytic jaundice, hemolytic anemia, and cholelithiasis symptoms were considerably alleviated after surgery (Table 1). Although this type of problem is uncommon, our observations revealed that the patient’s clinical symptoms had been positively relieved by the combination treatment.

**Table 1:** Comparison of the test results before and after the patient’s surgical treatment

	Preoperative	One-week postoperative
Red blood cells	2.09 × 10^12^/l	3.44 × 10^12^/l
Hemoglobin	67 g/l	106 g/l
Total bilirubin	139.4 μmol/l	24.2 μmol/l
Direct bilirubin	23.6 μmol/l	13.6 μmol/l

In summary, the management of cholelithiasis and significant splenomegaly associated with HS should be thoroughly evaluated considering the patient’s specific clinical symptoms and consequences. The combination of splenectomy and cholecystectomy is a sensible and efficient strategy with which to treat the patient and can enhance their overall prognosis. Due to the weakened immune functionality of patients after splenectomy, there are risks of infection, bleeding, and other complications. Therefore, to prevent infections, pneumococcal vaccines are often administered preoperatively, and penicillin is used postoperatively, though this does not eliminate the risk of infection. Over recent years, partial laparoscopic splenectomy has garnered significant attention. Stoehr *et al*. [6] performed a follow-up study of partial splenectomy (up to 6 years of follow-up) and showed that preserving as little as 10 cm^3^ of splenic tissue could effectively control hemolysis while avoiding postoperative immune decline and infections caused by total splenectomy. However, there remains a risk of recurrence, and some patients may require subsequent total splenectomy due to splenic regeneration [7]. Consequently, the optimal surgical approach for HS complicated by massive splenomegaly and gallstones still requires further exploration. The most suitable surgical plan should be selected based on the patient’s specific condition.
